# Therapy Persistence and Dose Escalation of Advanced Treatments in First and Second Line of Ulcerative Colitis and Crohn’s Disease – A Retrospective Cohort Analysis of Healthcare Claims Data

**DOI:** 10.1055/a-2836-5222

**Published:** 2026-04-21

**Authors:** Axel Dignass, Niels Teich, Stephan Kaiser, Juliane Sünwoldt, Evelyn Reinhard, Julia Borchert, Robert Kudernatsch

**Affiliations:** 1 Medical Clinic I 84491 Agaplesion Markus HospitalFrankfurt am MainGermany; 2 Internal Medicine Group Practice for Digestive and Metabolic DiseasesLeipzigGermany; 3 54374 Takeda Pharma Vertrieb GmbH & Co KGBerlinBEGermany; 4 633584 WIG2 GmbHLeipzigGermany

**Keywords:** Intestine, IBD, Statistics, Darm, CED, Statistik

## Abstract

**Background:**

There is limited data on advanced therapy outcomes in second-line vs. first-line advanced treatment of patients with ulcerative colitis (UC) and Crohn’s disease (CD).

**Methods:**

We conducted a retrospective health claims study of patients with UC and CD in Germany. We analyzed claims data from 2014 to 2021, and compared therapy persistence and dose escalation in first- (1L) and second-line (2L) advanced therapy. Our analysis included the approved therapies adalimumab (ADA), infliximab (IFX), ustekinumab (UST) and vedolizumab (VDZ) for both UC and CD patients and golimumab (GOL) and tofacitinib (TOF) for UC patients.

**Results:**

2,948 patients were included in the study and initiated 1L advanced therapy. Of these, 823 patients started a 2L advanced therapy. Time on treatment was generally shorter in 2L compared to 1L: In UC patients, persistence rates at 2 years ranged from 52.5% to 71.9% for 1L, and from 40.1% to 69.1% for 2L. In CD patients, persistence rates ranged from 66.6% to 86.6% for 1L, and from 59.9% to 74.3% for 2L. We observed more frequent dose escalations in 2L than in 1L for all advanced therapies except GOL in UC patients.

**Conclusions:**

Our study indicates that advanced therapies have shorter persistence and require higher doses when used as 2L treatment compared to 1L treatment. Future studies on reliable response predictors in patients with UC or CD receiving advanced treatments are likely to improve the selection of more efficacious 1L therapy.

## Zusammenfassung

**Hintergrund:**

Vergleichende Daten zum Therapieverlauf fortgeschrittener Therapien in der Erst- und Zweitlinie bei Colitis ulcerosa (UC) und Morbus Crohn (CD) sind bislang nur begrenzt verfügbar.

**Methoden:**

Wir führten eine retrospektive Analyse deutscher Krankenkassendaten (2014–2021) bei Patienten mit UC und CD durch und verglichen die Therapiepersistenz sowie die Dosissteigerung in der Erst- (1L) und Zweitlinie (2L). Unsere Analyse umfasste Adalimumab (ADA), Infliximab (IFX), Ustekinumab (UST), Vedolizumab (VDZ), Golimumab (GOL) und Tofacitinib (TOF).

**Ergebnisse:**

In die Studie wurden 2948 Patienten eingeschlossen, die eine fortgeschrittene Therapie begonnen hatten. Davon wechselten 823 Patienten in eine Zweitlinientherapie. Die Behandlungsdauer war bei 2L kürzer als bei 1L: Bei UC-Patienten lagen die Persistenzraten nach zwei Jahren zwischen 52,5% und 71,9% für 1L und zwischen 40,1% und 69,1% für 2L. Bei CD-Patienten lagen die Raten zwischen 66,6% und 86,6% für 1L und zwischen 59,9% und 74,3% für 2L. Wir beobachteten häufigere Dosissteigerungen in 2L im Vergleich zu 1L bei allen Therapien, mit Ausnahme von GOL bei UC-Patienten.

**Schlussfolgerungen:**

Unsere Studie zeigt, dass fortgeschrittene Therapien in der Zweitlinie eine geringere Persistenz aufweisen und häufiger höhere Dosierungen erfordern als in der Erstlinie. Weitere Studien zu verlässlichen Prädiktoren des Therapieansprechens bei UC oder CD könnten die Auswahl einer wirksameren Erstlinientherapie verbessern.

## 1 Introduction

Inflammatory bowel disease (IBD), encompassing Crohn’s disease (CD) and ulcerative colitis (UC), are chronic, relapsing conditions that significantly impact patients’ quality of life 1 2 . Symptoms such as abdominal pain, diarrhea, and weight loss negatively affect patients’ physical and emotional health 2 3 4 . Unlike acute illnesses, IBD is a lifelong condition that requires continuous management involving long-term medication use, lifestyle adjustments, and, in some cases, surgical interventions 4 5 .

Despite significant IBD treatment advances 6 7 , achieving long-term remission remains a challenge. Many patients do not respond to initial therapy, while others lose responsiveness over time. Such drug resistance is influenced by several cellular and molecular mechanisms: genetic predispositions, such as single nucleotide polymorphisms (SNPs) in cytokine or Human Leukocyte Antigen genes, play a key role in determining therapeutic failure. Additionally, the dysregulation of inflammatory signaling pathways can contribute to a diminished response 8 . Personalized treatment strategies including optimal sequence choices for therapy could be instrumental in overcoming these challenges 9 10 . However, data comparing therapy persistence between first-line advanced treatment and later treatment lines are scarce, particularly in real-world settings.

Our study addresses this gap by analyzing claims data in Germany from 2014 to 2021, focusing on therapy persistence and dose escalation of first-line (1L) and second-line (2L) advanced therapies in both UC and CD patients. By examining the approved advanced therapies—the monoclonal antibodies adalimumab (ADA), infliximab (IFX), ustekinumab (UST), vedolizumab (VDZ), golimumab (GOL), and the janus kinase inhibitor tofacitinib (TOF)—we aim to provide insights into the real-world application of these treatments and their impact on patient management.

## 2 Methods

### 2.1 Database

We conducted a non-interventional longitudinal retrospective study using health claims data from the WIG2 benchmark database. The database contains anonymized data from approximately 4.5 million patients insured by one of several different German statutory health insurance providers (SHIs). In Germany, health insurance is mandatory for all residents. Approximately 73 million people (90% of the population) are covered by SHI 11 . The WIG2 database is representative of the German SHI population regarding age, gender and morbidity 12 .

We analyzed data from 01 January 2014 to 31 December 2021 using codes documented for billing purposes. We analyzed documented inpatient and outpatient diagnoses (defined by the International Statistical Classification of Diseases and Related Health Problems 10 ^th^ Revision, German version; ICD-10 GM codes), prescribed medications documented by Anatomical Therapeutic Chemical (ATC) code, medical aids and operating procedures, and the temporal order of these diagnoses/events.

Since all data were anonymized according to German data protection regulations, their use for scientific purposes was in conformity with German law, and no additional permissions were needed from an institutional review board (IRB) or an independent ethics committee (IEC).

### 2.2 Study Population

The patient population under consideration is described in detail by Dignass et al. 13 . In brief, we selected advanced therapy-naive UC and CD patients (≥ 18 years) with at least one advanced IBD therapy prescription (ADA, IFX, TOF, GOL, UST, or VDZ) from 01 January 2015 to 30 June 2021 (index period). We defined the index date as the date of the first prescription with a UC or CD diagnosis (see **Supplementary Table 1** ) documented in the same quarter. We excluded patients who had received one of the advanced IBD therapies in the 12 months prior to the index date, as well as patients who could not be followed up with for at least six months after the index date (unless deceased). Thus, the total study period spanned from 01 January 2014 to 31 December 2021.

We observed advanced treatment-naïve patients from the start of their index therapy, which we defined as their first-line advanced therapy (1L), up until censoring. Censoring events included death, end of data availability (i.e., 31 December 2021), or end of insurance. To compare different therapy lines, we evaluated any switch to a subsequent advanced therapy (2L, 3L). This switch was defined as a prescription of another advanced therapy without a concomitant refill prescription of the previous therapy within a 180-day period after consumption of defined daily dose (DDD) supply. The individual therapy lines were defined by the chronological order (1L, 2L, or 3L) of the advanced therapy agents.

### 2.3 Study endpoints and analysis

A detailed overview of the outcome variables and their definitions is summarized in **Supplementary Table 2** .

We assessed age, gender, and IBD-related hospitalizations (number of hospitalization events and duration of hospital stay) for each patient in the pre-index year (baseline characteristics).

Outcomes of interest included persistence (time on treatment, ToT), and the proportion of patients experiencing inadequate responses (dose escalation, IBD-related hospitalizations and surgeries) for the advanced therapy lines (1L or 2L) in UC- and CD-patients, respectively.

Time on treatment (ToT) was defined as the number of days from the start of one advanced therapy line (either 1L or 2L) to the start of the next advanced therapy line (2L or 3L, respectively), or censoring date minus the number of days without treatment.

Dose escalation was defined as an increase of more than 1.5 times the recommended dose during the maintenance phase over the course of 3 consecutive prescription intervals (following the respective European Summary of Product Characteristics as of October 2022). We assumed the maintenance phase began after the drug-specific induction period: 4 weeks for ADA, 6 weeks for GOL, 14 weeks for IFX and VDZ, 8 weeks for TOF, and 12 weeks for UST.

We derived the number of IBD-related hospitalizations and surgeries from IBD-related ICD-10 codes and procedure codes as specified in **Supplementary Table 1** .

Descriptive analyses were conducted in this study by disease type (UC or CD), treatment subgroup (ADA, IFX, TOF, GOL, UST, or VDZ) and advanced therapy line (1L or 2L). We did not perform statistical tests to compare different treatment subgroups.

We described baseline patient characteristics using mean, standard deviation and median, as appropriate.

Cumulative rates for time on treatment (ToT) were derived from unadjusted Kaplan-Meier analyses. The final Kaplan-Meier estimate was calculated by multiplying the successive probabilities by all previously calculated probabilities 14 . We reported the mean and median, and the comparative outcome at the 2-year timepoint.

We reported dose escalation, IBD-related hospitalizations and surgeries by the number and proportion of patients experiencing these inadequate responses.

Data management and analysis was performed using Microsoft structured query language (SQL) Server 2016 and R Version 4.1.

### 2.4 Usage of AI Tools

During the preparation of this manuscript, we used OpenAI ChatGPT-4 to enhance writing style and check grammar and spelling. We subsequently reviewed, edited and approved all content.

## 3 Results

### 3.1 Patient population and characteristics

The patient population under consideration and advanced treatment sequences and frequencies are described in detail by Dignass et al 13 . In brief, a total number of 2,948 patients (UC: 1,157; CD: 1,791) were included in the study and constitute the number of patients at the start of the first therapy line (1L). Of these, 823 patients (UC: 406; CD: 417) received a second-line advanced therapy, and 259 (UC: 154; CD: 105) of them subsequently switched to a third-line treatment. Due to the low number of patients per treatment group in third-line therapy, our descriptive analysis focused on therapy persistence and dose escalation in first and second line only.

In the UC patient group, 59% of patients were male and we observed a higher proportion of males in all treatment groups except UST (14 females vs. 11 males). The median age of all patients ranged between 37.0 and 53.0 years ( Table 1 ). Across genders, the youngest patients were female, treated with GOL at index (mean 38.2 years), the oldest were female patients treated with UST (mean 48.5 years). Before starting advanced therapy, UC patients beginning with IFX treatment had the highest number of IBD-related hospitalizations (0.53 events per patient year) with a mean duration of 4.6 days in hospital care. We observed the lowest number of hospitalizations in the GOL group (0.18 events per patient year) with a mean duration of 1.8 days of hospital stay ( Table 1 ).

**Table:** **Table 1** Age and gender distribution at start of first-line advanced therapy, and IBD-related hospitalization of ulcerative colitis patients in the year before start of first-line advanced therapy. Mean, SD and median of age by gender and treatment. Mean and SD of hospitalization events and duration of hospital-stay for total patients per treatment group.

Treatment at index		Number of patients	Age at start of first-line advanced therapy (years)			Number of hospitalizations within one year before first-line advanced therapy		Duration of hospital stay (days)	
			Mean	SD	Median	Mean	SD	Mean	SD
**Adalimumab**									
	*female*	139	**41.7**	13.7	41.0	**0.36**	0.70	**3.3**	8.1
	*male*	198	**44.6**	12.7	46.0				
**Golimumab**									
	*female*	50	**38.2**	12.7	38.0	**0.18**	0.43	**1.8**	5.2
	*male*	61	**44.9**	15.4	45.0				
**Infliximab**									
	*female*	166	**40.3**	14.5	37.0	**0.53**	0.96	**4.6**	8.8
	*male*	253	**41.8**	15.0	41.0				
**Tofacitinib**									
	*female*	7	**47.6**	18.3	48.0	**0.29**	0.59	**2.4**	5.3
	*male*	10	**45.5**	14.7	46.0				
**Ustekinumab**									
	*female*	14	**48.5**	18.9	53.0	**0.48**	0.77	**5.0**	10.3
	*male*	11	**42.2**	15.4	41.0				
**Vedolizumab**									
	*female*	104	**43.2**	14.8	40.0	**0.40**	0.79	**3.3**	7.2
	*male*	144	**46.6**	16.1	45.0				

In the CD patient group, we observed a higher proportion of female patients in all treatment groups ranging from 51% to 56%. The mean age varied between 37.6 years (males, IFX treatment) and 48.6 years (males, VDZ treatment) and the median ranged from 36.0 to 51.0 years ( Table 2 ). For the CD patient group, the number of hospitalizations before index was also highest in patients receiving IFX (0.69 events per patient year) with a mean duration of 6.2 days in hospital care. Patients starting UST had the lowest number of IBD-related hospitalizations in the 12 months prior to advanced therapy start (0.41 events) with a mean duration of 3.2 days in hospital ( Table 2 ).

**Table:** **Table 2** Age and gender distribution at start of first-line advanced therapy and IBD-related hospitalization of Crohn’s disease patients in the year before start of first-line advanced therapy. Mean, SD and median of age by gender and treatment. Mean and SD of hospitalization events and duration of hospital-stay for total patients per treatment group.

Treatment at index		Number of patients	Age at start of first-line advanced therapy (years)			Number of hospitalizations within one year before first-line advanced therapy		Duration of hospital stay (days)	
			Mean	SD	Median	Mean	SD	Mean	SD
**Adalimumab**									
	*female*	520	**38.6**	13.2	38.0	**0.49**	0.74	**4.2**	12.8
	*male*	403	**39.4**	13.5	39.0				
**Infliximab**									
	*female*	313	**39.0**	15.2	38.0	**0.69**	0.95	**6.2**	12.7
	*male*	303	**37.6**	13.2	36.0				
**Ustekinumab**									
	*female*	62	**46.5**	15.0	44.5	**0.41**	0.74	**3.2**	6.6
	*male*	51	**41.5**	15.3	41.0				
**Vedolizumab**									
	*female*	77	**43.9**	15.3	42.0	**0.46**	0.75	**5.4**	13.9
	*male*	62	**48.6**	16.7	51.0				

### 3.2 Therapy persistence and dose escalation

#### 3.2.1 Ulcerative colitis

**Fig. 1:**
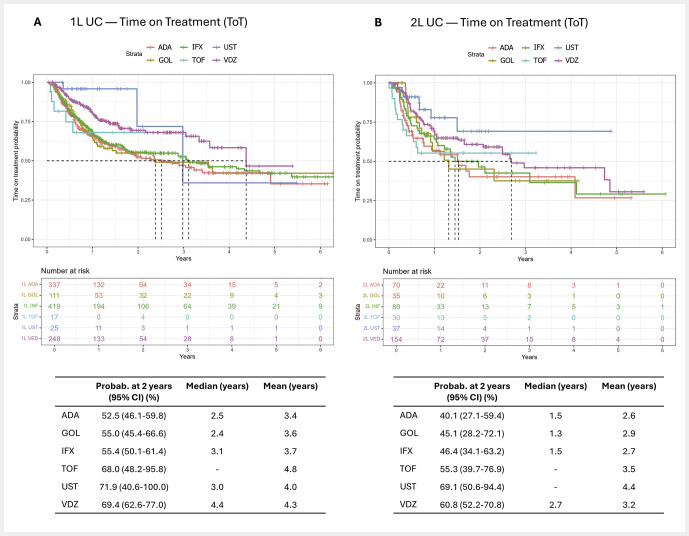
Unadjusted Kaplan-Meier curves for time on treatment (ToT) in first-line ( **A** ) and second-line ( **B** ) advanced therapy of ulcerative colitis (UC) patients. Median and mean, and comparative outcome at the 2-year timepoint is shown. The median cannot be calculated if the ToT probability is > 0.5. ADA: Adalimumab; GOL: Golimumab; IFX: Infliximab; TOF: Tofacitinib; UST: Ustekinumab; VDZ: Vedolizumab; 1L: first-line advanced treatment; 2L: second-line advanced treatment. Censored survival times and median time to event are marked in the Kaplan-Meier plot.

In UC patients, the probability of consistent therapy (time on treatment, ToT) differed between the first-line (1L) and second-line (2L) advanced therapy: First-line advanced therapy persistence rate at 2 years ranged from 52.5% to 71.9% and was highest in UST (71.9%), VDZ (69.4%) and TOF (68.0%) ( Fig. 1 A). In the second line, the percentage of patients with consistent advanced therapy at 2 years was lower compared to 1L and ranged from 40.1% to 69.1% with highest persistence rate in UST (69.1%) and VDZ (60.8%) ( Fig. 1 B). It is important to note that patient numbers were low in the TOF and UST treatment groups.

We observed dose escalation more frequently in 2L than in 1L therapy for all advanced treatments except GOL. The total increase was most pronounced for UST (1L: 8.0%; 2L: 32.4%) and IFX (1L: 16.5%; 2L: 33.8%). Patients who received VDZ had the lowest dose escalation rates in both treatment lines (1L: 1.2%; 2L: 8.4%). We observed a strong increase in IBD-related hospitalizations for TOF only, rising from no events in 1L to 20% in 2L. The proportion of IBD-related surgeries did not differ much between 1L and 2L advanced therapy ( Table 3 ).

**Table:** **Table 3** Number (N) and proportions (%) of ulcerative colitis patients with inadequate response in first-line (1L) and second-line (2L) advanced therapy.

Treatment		Total number	Dose escalation		IBD-related hospitalization		IBD-related surgery	
			N	%	N	%	N	%
**1L**								
	*Adalimumab*	337	44	**13.1%**	40	**11.9%**	23	**6.8%**
	*Golimumab*	111	39	**35.1%**	13	**11.7%**	10	**9.0%**
	*Infliximab*	419	69	**16.5%**	86	**20.5%**	48	**11.5%**
	*Tofacitinib*	17	2	**11.8%**	0	**0.0%**	1	**5.9%**
	*Ustekinumab*	25	2	**8.0%**	2	**8.0%**	3	**12.0%**
	*Vedolizumab*	248	3	**1.2 %**	25	**10.1%**	23	**9.3%**
**2L**								
	*Adalimumab*	70	15	**21.4%**	11	**15.7%**	5	**7.1%**
	*Golimumab*	35	10	**28.6%**	5	**14.3%**	2	**5.7%**
	*Infliximab*	80	27	**33.8%**	12	**15.0%**	13	**16.3%**
	*Tofacitinib*	30	7	**23.3%**	6	**20.0%**	1	**3.3%**
	*Ustekinumab*	37	12	**32.4%**	5	**13.5%**	5	**13.5%**
	*Vedolizumab*	154	13	**8.4%**	25	**16.2%**	23	**14.9%**

#### 3.2.2 Crohn’s disease

**Fig. 2:**
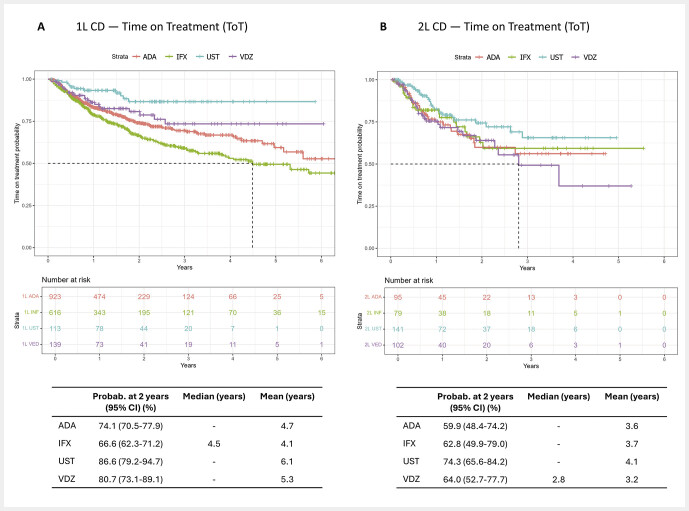
Unadjusted Kaplan-Meier curves for time on treatment (ToT) in first-line ( **A** ) and second-line ( **B** ) advanced therapy of Crohn’s disease (CD) patients. Median and mean, and comparative outcome at the 2-year timepoint is shown. The median cannot be calculated if the ToT probability is > 0.5. ADA: Adalimumab; IFX: Infliximab; UST: Ustekinumab; VDZ: Vedolizumab; 1L: first-line advanced treatment; 2L: second-line advanced treatment. Censored survival times and median time to event are marked in the Kaplan-Meier plot.

Therapy persistence in CD patients was generally high: ToT probability did not drop below 0.5 except for IFX in 1L after 4.5 years and for VDZ in 2L after 2.8 years ( Fig. 2 ). The first-line therapy persistence rate at 2 years ranged from 66.6% to 86.6% and was highest in UST (86.6%) and VDZ (80.7%) ( Fig. 2 A). In the second line, the percentage of patients who remained on the advanced therapy at 2 years was lower compared to 1L and ranged from 59.9% to 74.3% with highest persistence rate in UST (74.3 %) ( Fig. 2 B).

We observed dose escalation more frequently in all 2L advanced therapies than in 1L therapies. The total increase was most prominent for ADA (1L: 11.3%; 2L: 24.2%) and IFX (1L: 18.2%; 2L: 29.1%). Patients receiving VDZ had the lowest dose escalation rates in both treatment lines (1L: 0.7%; 2L: 5.9%). We observed a slight increase in IBD-related hospitalization rates for UST (1L: 14.2%; 2L: 20.6%) and VDZ (1L: 12.9%; 2L: 19.6%). IBD-related surgery rates did not differ much between 1L and 2L advanced therapy ( Table 4 ).

**Table:** **Table 4** Number (N) and proportions (%) of Crohn’s disease patients with inadequate response in first-line (1L) and second-line (2L) advanced therapy.

Treatment		Total number	Dose escalation		IBD-related hospitalization		IBD-related surgery	
			N	%	N	%	N	%
**1L**								
	*Adalimumab*	923	104	**11.3%**	166	**18.0%**	185	**20.0%**
	*Infliximab*	616	112	**18.2%**	145	**23.5%**	136	**22.1%**
	*Ustekinumab*	113	28	**24.8%**	16	**14.2%**	22	**19.5%**
	*Vedolizumab*	139	1	**0.7%**	18	**12.9%**	25	**18.0%**
**2L**								
	*Adalimumab*	95	23	**24.2%**	20	**21.1%**	18	**18.9%**
	*Infliximab*	79	23	**29.1%**	18	**22.8%**	19	**24.1%**
	*Ustekinumab*	141	39	**27.7%**	29	**20.6%**	27	**19.1%**
	*Vedolizumab*	102	6	**5.9%**	20	**19.6%**	16	**15.7%**

## 4 Discussion

Our study provides significant insights into advanced therapy persistence and dose escalation for patients with UC and CD. We observed that 2L advanced treatments generally lead to shorter therapy persistence compared to first-line advanced treatments. Specifically, in UC patients, the persistence rate at 2 years ranged from 52.5% to 71.9% for 1L and from 40.1% to 69.1% for 2L. For CD patients, the 2-year persistence rate ranged from 66.6% to 86.6% for 1L and from 59.9% to 74.3% for 2L. These findings underscore the importance of selecting the optimal 1L advanced therapy to enhance patient outcomes.

When comparing our results with existing literature, we find a consistent trend across several studies reporting reduced persistence in later lines of advanced therapy. For instance, Zhao et al. 15 and Koo et al. 16 align with our findings, showing lower persistence rates in 2L treatments. Kapizioni et al. 17 examined the effectiveness of VDZ in UC and CD patients, and UST in CD patients registered in the UK IBD BioResource platform. Significant differences between treatment lines (1L, 2L, and 3L) were observed for VDZ in CD patients only, with effectiveness highest in 1L and declining in subsequent therapy lines.

However, not all studies confirm this trend. The ROTARY 18 19 and RECORDED 20 studies present contrasting findings, and report higher persistence in 2L advanced treatments. These discrepancies may arise from differences in study populations, designs, or definitions of persistence. Additionally, variations in healthcare systems, patient demographics, or adherence levels could influence these outcomes. By examining these differences, our study adds to the ongoing debate about therapy sequencing and persistence, emphasizing the need for context-specific strategies.

In our study, dose escalation was more frequent in 2L advanced therapies, indicating a need for higher doses to maintain therapeutic efficacy. In UC, IFX showed a relevant increase in dose escalation from 16.5% in 1L to 33.8% in 2L. Similarly, the UST dose escalated considerably more often in 2L with 32.4%, compared to 8.0% in 1L. In CD patients, ADA and IFX demonstrated notable increases in dose escalation rates in 2L treatments, while UST dose escalation was generally high in 1L and 2L (24.8% vs 27.7%).  These patterns may suggest that maintaining disease control is more challenging as patients progress to later lines of advanced therapy. Increases in dose escalation call for careful monitoring and potential adjustments in treatment strategies to manage disease progression effectively. Yet, this also highlights that even with increased dosing in 2L, persistence remains considerably decreased in 2L, i.e. more intense treatment results in less effectiveness. Moreover, off-label dosages should be avoided in such situations.

IBD-related surgery rates were largely comparable between treatment lines in both UC and CD patients. An increase in IBD-related hospitalizations in 2L compared to 1L was observed for only a limited number of treatments. Generally, it is important to keep in mind that claims data do not allow for an assessment of an individual patient’s disease history or disease severity. Consequently, it is not possible to determine whether observed increases in dose escalation or hospitalization rates are attributable to treatment choice itself or reflect underlying differences in disease severity or other confounders. Future studies are needed to identify reliable predictors of response to advanced treatments in IBD patients, which could help optimize 1L advanced therapy selection and improve long-term outcome.

### 4.1 Limitations

This study has some limitations that should be considered when interpreting the findings. Firstly, due to small sample sizes for certain treatment groups, specifically TOF and UST, and the unadjusted nature of the comparisons, the reliability and significance of the results are subject to uncertainty. This can be attributed to statistical fluctuations, distorting confounding factors, and selection bias. Since we relied solely on descriptive comparisons, without performing statistical tests to evaluate differences between treatment subgroups, the findings should be interpreted only as indicative trends rather than definitive or generalizable conclusions.

Secondly, IBD is characterized by alternating periods of flares and remission. However, health claims data do not include clinical information on disease severity or activity at baseline or during treatment, which limits the ability to directly compare the effectiveness of different therapies.

Additionally, retrospective claims data analyses are inherently limited by the framework of the German reimbursement system. In hospital settings, treatments are billed as lump sums, capturing diagnoses and procedures while typically incorporating the costs of administered drugs. Only high-cost medications with special reimbursement codes – such as ADA, IFX, GOL, UST, and VDZ – are documented separately. As a result, the usage of certain drugs, like TOF or systemic steroids administered during inpatient stays, may be underestimated.

Finally, in outpatient settings, billing practices further complicate precise data interpretation. Each physician documents all patient visits as a single case for billing purposes at the end of each quarter. This system makes it impossible to accurately track the timing of individual diagnoses within that time frame, potentially obscuring the sequence of events. Prescription dates, however, can sometimes provide clarity regarding the order of clinical developments.

## 5 Conclusion

In our real-world observational study, we found that advanced therapies tend to have reduced effectiveness when employed as 2L treatments compared to 1L options. This is evidenced by shorter treatment durations and increased rates of dose escalation during second-line advanced therapy for both UC and CD patients. Future studies on reliable predictors of response to advanced treatments in IBD patients are likely to improve the potential of effective 1L therapy.

## 6 Data sharing and availability

The datasets generated and/or analyzed during this study are not publicly available due to data protection aspects. The data underlying this article will be shared at aggregate/population level upon reasonable request to the corresponding author.

## 7 List of non-standard abbreviations

Inflammatory Bowel Disease (IBD)

Ulcerative colitis (UC)

Crohn’s disease (CD)

Adalimumab (ADA)

Infliximab (IFX)

Golimumab (GOL)

Tofacitinib (TOF)

Ustekinumab (UST)

Vedolizumab (VDZ)

Anatomical Therapeutic Chemical (ATC)

Time on treatment (ToT)

First-line therapy (1L)

Second-line therapy (2L)

## Supplementary Material

Supplementary Material Zusatzmaterial
