# Supplementary material for: Therapy Persistence and Dose Escalation of Advanced Treatments in First and Second Line of Ulcerative Colitis and Crohn’s Disease – A Retrospective Cohort Analysis of Healthcare Claims Data
Source: Z Gastroenterol. 2026 Apr 21;64(6):680–8. doi: 10.1055/a-2836-5222 (PMC13286670; doi:10.1055/a-2836-5222)
Supplement: Supplementary file 1 — Supplementary Material [file 10-1055-a-2836-5222_28365678.pdf]

Supplementary Table 1:

Codes for CD and UC diagnoses, and IBD- related complications and surgeries

Code	Description
**CD and UC patient identification**	
ICD-10 K50.0	Crohn disease of small intestine
ICD-10 K50.1	Crohn disease of large intestine
ICD-10 K50.8	Other Crohn disease
ICD-10 K50.9	Crohn disease, unspecified
ICD-10 K51.0	Ulcerative (chronic) pancolitis
ICD-10 K51.2	Ulcerative (chronic) proctitis
ICD-10 K51.3	Ulcerative (chronic) recto sigmoiditis
ICD-10 K51.4	Inflammatory polyps of colon
ICD-10 K51.5	Left sided colitis
ICD-10 K51.8	Other ulcerative colitis
ICD-10 K51.9	Ulcerative colitis, unspecified
**IBD-related complications**	
ICD-10 K61.-	Abscesses
ICD-10 H20.0	Acute and subacute iridocyclitis
ICD-10 K83.0	Cholangitis
ICD-10 L52.-	Erythema nodosum
ICD-10 L88.-	Pyoderma gangraenosum
ICD-10 K83.-	Primary Sclerosing Cholangitis
**IBD-related surgeries**	
OPS 5-45-	Intestinal incision, excision, resection, and anastomosis
OPS 5-46-	Other intestinal procedures
OPS 5-48-	Rectal procedures
OPS 5-49-	Anus procedures
OPS 5-98-	Surgical techniques and operations for special care situations.
EBM 31157, 36157	Intestinal incision, excision, resection, and anastomosis
EBM 31154, 36154	Other intestinal procedures
EBM 31175, 36175	Rectal procedures
EBM 31172, 36172	Anal procedures

ICD-10 = International Statistical Classification of Diseases and Related Health Problems 10th Revision

EBM = Einheitlicher Bewertungsmaßstab (Uniform valuation standard)

OPS = Operationen- und Prozedurenschlüssel (Operation and procedure code)

Supplementary Table 2:

Outcome variables and definitions

Outcome variable	Operational definition
**Treatment line**	Number of patients and frequency per line of advanced treatment stratified by diagnosis (UC/CD)
**ToT**	Number of days from start of one treatment line to start of next treatment line or censoring date minus the number of days without treatment. Outcomes are calculated according to Kaplan-Meier, i.e., the time until the first event per patient occurs
**Therapy switch**	Prescription of another advanced therapy without a concomitant refill prescription of the index therapy within a time frame of 180 days after consumption of defined daily dose [DDD] supply
**Dose escalation**	In patients for whom a maintenance phase was observable, dose escalation was defined as a >1.5 time-increase of the recommended dose of index therapy (following European Summaries of Product Characteristics) across 3 consecutive index therapy prescriptions. When maintenance therapy started was defined specifically to index drug: Maintenance therapy was assumed following the initiation phase, specified per drug: 4 weeks for ADA; 6 weeks for GOL; 14 weeks for IFX or VDZ; 8 weeks for TOFA; 12 weeks for UST.
**Number of IBD-related hospitalizations per patient year**	Inpatient patients with a main discharge diagnosis of UC/CD or IBD-related complications (Supplementary Table 1)
**Number of IBD-related surgeries per patient year**	For included inpatient and outpatient service and procedure codes see Supplementary Table 1
**Duration of IBD-related hospital stays per patient year**	Number of days from hospital admission to discharge with a primary diagnosis of UC/CD or IBD-related complications (Supplementary Table 1)
